# Outpatient Parenteral Antimicrobial Therapy for Infective Endocarditis—Model of Care

**DOI:** 10.3390/antibiotics12020355

**Published:** 2023-02-08

**Authors:** Dylan Rajaratnam, Rohan Rajaratnam

**Affiliations:** 1 Liverpool Hospital, Sydney, NSW 2170, Australia; 2 School of Medicine, Western Sydney University, Campbelltown Campus, Sydney, NSW 2560, Australia; 3 School of Medicine, Southwest Clinical School, The University of New South Wales, Sydney, NSW 2170, Australia

**Keywords:** infective endocarditis, OPAT, multidisciplinary team

## Abstract

Infective endocarditis (IE) is a serious infectious disease with significant mortality and morbidity placing a burden on healthcare systems. Outpatient antimicrobial therapy in selected patients has been shown to be safe and beneficial to both patients and the healthcare system. In this article, we review the literature on the model of care for outpatient parenteral antimicrobial therapy in infective endocarditis and propose that systems of care be developed based on local resources and all patients admitted with infective endocarditis be screened appropriately for outpatient antimicrobial therapy.

## 1. Introduction

Infective endocarditis (IE) is an infectious disease with an annual incidence of 3–10/100,000 with a mortality of up to 30% at 30 days [1,2], and approximately 40% at one-year [3] where, despite the improvements in modern medicine, the mortality has not improved in over 2 decades [2]. Morbidity from heart failure, severe valvular incompetence, structural destruction (abscess, perforation or fistula formation) and embolic or neurological complications are common and may require surgical intervention in conjunction with prolonged medical management [1,4,5,6].

The epidemiology of IE has gradually changed over the years within developed countries, with degenerative valve disease, diabetes, cancer, intravenous drug use and congenital heart disease replacing rheumatic heart disease as the major risk factors for infective endocarditis [7]. This has led to the average patient being older and frailer with increasing comorbidities [2]. Increasing use of long-term intravenous (IV) lines and invasive procedures (i.e., cardiac implantable electronic devices) has led to increased rates of staphylococcal bacteremia [7] and now healthcare-associated IE accounts for 25–30% of patients with IE [1,2]. Men are at 2–3 times greater risk of IE when compared to females [8].

Infective endocarditis requires a prolonged duration of therapy, often for a period of 4–6 weeks due to the density of bacteria within vegetations, low bacterial metabolic activity, production of protective biofilms on prosthetic material and frequently slow bactericidal activity of antimicrobial agents [2,9]. Due to the long duration of antimicrobial treatment, this can contribute to a large economic and resource burden on the healthcare system. Here, we review the literature for outpatient treatment of IE and comment on the safe delivery of this management for patients with IE suitable for outpatient parenteral antimicrobial therapy (OPAT).

## 2. Outpatient Parenteral Antibiotic Treatment (OPAT)

OPAT is the “administration of parenteral antimicrobials in an outpatient ambulatory setting” [10] and can result in significant benefit to both the healthcare system and patients. Therapy as an outpatient benefits the hospital system by reducing costs. In the United States, there are 40,000–50,000 new cases each year, with an average hospital cost in excess of USD 120,000 per patient [2]. Lacroix et al. demonstrated that using OPAT therapy to treat infective endocarditis can save over EUR 15,000 (USD 14,800) per patient and therefore minimizes the significant burden that IE places on the healthcare system [11]. Additionally, outpatient therapy reduces the burden on healthcare resources by reducing the length of inpatient stay and demand for limited healthcare resources.

Outpatient therapy also benefits patients by reducing exposure to nosocomial risks, such as hospital-acquired infections, venous thromboembolism and pressure injuries [10,12,13,14]. Extended hospital stays are known to reduce quality of life and extend the time for reintegration into everyday life [14], this was shown in a study focusing on patients discharged with outpatient management regimens, showing a significant improvement in physical functioning, pain and emotions [15].

We conducted a literature search of the Embase and Medline databases from January 2007 through November 2022, limited to publications in English, using the terms “infective endocarditis” AND “antibiotic” OR “antimicrobial” AND “outpatient” OR “OPAT” OR “home care”. The selection included clinical trials, observational studies, review articles and guidelines. We also reviewed reference articles cited in guidelines published by the American Heart Association (AHA) [3], European Society of Cardiology (ESC) [16] and the Working Party of the British Society for Antimicrobial Chemotherapy [17]. Studies published prior to 2007 that were considered pertinent to the review were also included (Table 1).

## 3. Building a Model of Care

### 3.1. Diagnosis

The American Heart Association (AHA) [3] suggests that patients with an unexplained fever for more than 48 h who are at risk of IE (valvular heart disease, prosthetic heart valves, certain congenital or heritable heart abnormalities, immunodeficiency states or intravenous drug users) or patients with newly diagnosed left-sided valve regurgitation should have at least two sets of blood cultures taken at separate times prior to antimicrobial initiation. The modified Duke criteria should be used to evaluate a patient with suspected IE, and a transthoracic echocardiogram (TTE) is recommended in patients with suspected IE to identify vegetations (a major criterion in the modified Duke criteria), to assess the severity of valvular lesions, ventricular function and pulmonary pressures and to screen for complications [3]. Transoesophageal echocardiogram (TEE) is recommended where IE is suspected and TTE is non-diagnostic (TEE has a positive predictive value in both native and prosthetic valve endocarditis of 90%), when complications are suspected or known, or when intracardiac device leads are present [3]. Nuclear molecular techniques are evolving as important methods of diagnosis in patients with diagnostic difficulties and classified as “possible IE” using the Duke criteria. Single-photon emission computed tomography (SPECT), which utilizes autologous radiolabelled leucocytes and positron emission tomography (PET) imaging utilizes ^18^F-FDG (^18^F-Fludeoxyglucose), which is incorporated into activated leucocytes, monocytes, macrophages and CD4^+^ T-lymphocytes, is being increasingly used to reduce the rate of misdiagnosed IE and for the detection of peripheral embolic and metastatic infectious events [16].

### 3.2. Multidisciplinary Infective Endocarditis Team

A multidisciplinary team is vital in the management of infective endocarditis, and this consists of cardiologists and cardiac surgeons to provide guidance in diagnosis, investigation and clinical management; infectious disease specialists and/or microbiologists to provide expertise in the identification of the causative organisms and to direct the choice and duration of antimicrobial therapy; anaesthesiologists for peri- and intraoperative diagnosis and management; and there should also be access to neurologists and neurosurgical expertise, as up to 30% of patients will experience symptomatic neurological events. In certain circumstances, patients may need congenital heart disease specialists [1,2,3]. The pharmacist is involved in antibiotic counselling, supply and therapeutic drug monitoring (e.g., aminoglycosides, glycopeptides) and a clinical nurse specialist organizes the logistics behind the program, including vascular access selection, staffing and directly communicating with patients. Finally, nurses are involved with the day-to-day care of patients, administering antimicrobials, taking vitals and an ECG when indicated [4,10,13,19,23].

The AHA strongly recommends that patients should be managed in centres with immediate access to cardiothoracic surgery during the initial observation stages of IE, given that the patients may require urgent surgical intervention [3]. Uncomplicated IE can normally be managed locally with close communication with the infective endocarditis team [1]; however, rapid transfer to a hospital with cardiothoracic surgical facility should be available if the need arises.

In Italy [20], a formalized multidisciplinary team consisting of a cardiologist, infectious disease specialist, microbiologist and cardiac surgeon evaluating patients within 12 h of admission, identifying those requiring early surgery within 48 h and monitoring stable patients weekly, had a significant effect on outcomes in patients with native valve endocarditis. A study comparing the outcomes of patients before and following the introduction of the multidisciplinary team demonstrated a reduction in overall in-hospital mortality (28% vs. 13%, *p* = 0.02), mortality of surgery during the active phase (47% vs. 13%, *p* = 0.001), and 3-year mortality (34% vs. 16%, *p* = 0.0007) despite patients being older (mean age 54.2 vs. 59.1, *p* = 0.01) and having more co-morbidities (Charlson index 2.31 vs. 3.01, *p* = 0.02).

### 3.3. Initial Stabilization

Infective endocarditis has significant morbidity and mortality, where the first two weeks after diagnosis is the period of highest complication rate, and therefore an initial inpatient stabilization period is recommended [4,16,24]. The most significant adverse prognostic factors in IE are old age, heart failure, paravalvular complications, stroke, prosthetic valve endocarditis and infection with *Staphylococcal aureus* [7]. The risk of embolism is highest during the first days after initiation of antibiotic treatment and decreases after two weeks [7].

Two weeks of in-hospital antimicrobial management is particularly recommended with staphylococcal IE due to its higher rates of septic metastasis and embolic events [12,13]. This is supported by the 2015 European Society of Cardiology (ESC) guideline [16], which identifies two different phases during the course of antibiotic therapy, a first critical phase of 2 weeks during which OPAT has restricted indication and a second, continuation phase beyond 2 weeks where OPAT may be feasible. A prospective single-centre study conducted in Barcelona by Cervera et al. [12] provides evidence that in certain groups, OPAT may be initiated earlier. The study included 392 consecutive episodes of IE who were admitted to the Hospital Clinic of Barcelona OPAT program from 1997 to 2006. In total, 32 patients had *Streptococcus gallolyticus* or viridans group streptococcus (VGS) (22 native valve endocarditis and 9 prosthetic valve endocarditis). These patients received an intravenous course of 7–10 days followed by initiation into an OPAT program. Four patients required readmission to hospital and there were no mortalities. Therefore, it may be feasible for patients with native valve *Streptococcus gallolyticus* or VGS IE to have OPAT after one week of in-hospital antimicrobial management, however more research is required.

### 3.4. Patient Selection and Exclusion

Careful patient selection into an OPAT program is critical to minimize treatment failure and complication rates (Table 2). Patients contraindicated to OPAT are those with IE complications, such as heart failure, renal failure, septic shock, neurological complications, or those who participate in active illicit drug use [12,25]. The guideline for the management of infective endocarditis by The Working Party of the British Society of Antimicrobial Chemotherapy (BSAC) by Gould et al. [17] recommends the consideration of OPAT in those who are stable and responding well to therapy, without signs of heart failure, without uncontrolled extracardiac foci of infection and without any of the indications for surgery, such as aortic or mitral IE with severe acute regurgitation or fistula causing refractory pulmonary oedema/shock. They also recommend excluding locally uncontrolled infection (abscess, false aneurysm, enlarging vegetation, persisting fever and positive blood culture for ≥10 days) and infection caused by fungi or multi-resistant microorganisms or vegetations likely to embolize (aortic or mitral IE with vegetations >10 mm with complications or large vegetations >15 mm).

High-risk patients (elderly, prosthetic valve endocarditis, multiple patient comorbidities) and those with high-risk culprit organisms (*Staphylococcal aureus*, *fungi* and *non-HACEK Gram-negative bacilli*) require careful consideration prior to outpatient therapy [1]. Patients should also be assessed for treatment complications prior to OPAT therapy, being screened for adverse drug effects, diarrhoea, nausea, vomiting and catheter line infections [12,25].

For patients to be considered into an OPAT program they must have adequate cognitive function and stable mental health, with access to outpatient healthcare services such as clinics/HITH (hospital-in-the-home) services and have access to transport when required. Patients should be clinically stable with signs of treatment response (negative blood cultures (three days), apyrexial (seven days), decreasing neutrophil count, decreasing c-reactive protein (CRP) level, have a stable IV access and stable renal and hepatic function [4,19,21].

It is recommended that patients have an electrocardiogram (ECG) to ensure the absence of conduction block (2nd and 3rd degree atrioventricular block) and an echocardiogram confirming a decrease in the size of vegetations since the start of antimicrobial therapy, vegetations being ≤10 mm and the absence of paravalvular complications [4,17].

### 3.5. Models of Delivery

OPAT can be administered in multiple different ways [4,10,12] either through an outpatient clinic or ambulatory care setting, or via home visit treatment or self-administration. OPAT through an outpatient clinic/ambulatory care setting is very common, involving a peripherally inserted central catheter (PICC), with patients presenting to the healthcare service, being monitored for symptoms or signs of complications and having vital signs taken and laboratory investigations or an ECG taken if indicated. Patients receive their therapy by appropriately trained nursing staff.

Home visit treatment involves administration of antibiotics via a PICC within a patient’s place of residence (hospital in the home). This requires daily visits by appropriately trained nursing staff, with patients being monitored for symptoms or signs of complications and having vital signs taken. This cohort must be monitored closely with a low threshold to refer to the hospital.

Finally, self-administration treatment is where patients self-administer their antibiotics through a PICC or orally if utilizing a hybrid intravenous/oral regimen. This cohort must have strict patient selection with patients having direct access/contact with the OPAT team. This cohort must be reviewed regularly in an outpatient clinic/ambulatory care setting to conduct necessary monitoring of symptoms or signs of complications, laboratory investigations and an ECG where required.

### 3.6. Hybrid Intravenous/Oral Regimen

Oral antimicrobial stepdown regimens after a period of intravenous therapy have been increasingly utilized for the management of infective endocarditis. A few small trials over the years have shown the efficacy of a hybrid intravenous/oral regimen (initial period of intravenous antibiotics followed by oral antibiotics) [26,27,28]. Recently, the Partial Oral versus Intravenous Antibiotic Treatment of Endocarditis (POET) trial [22] was carried out, which was a randomized non-inferiority multicentre trial conducted in Denmark. This trial had 400 selected patients with stable left-sided *Streptococcus spp.*, *Enterococcus faecalis*, *Staphylococcus aureus* or *coagulase-negative staphylococci* infections. All patients initially received at least 10 days of intravenous antibiotics, had a satisfactory response to treatment, were afebrile for at least 48 h, had a c-reactive protein less than 25% of the peak level or less than 20 mg/L, a leucocyte count less than 15 × 10^9^/L and a transesophageal echocardiogram showing no abscess formation or other indications for surgery. Patients were randomized to ongoing intravenous therapy (199 patients) or stepped down to oral antimicrobials (201 patients). Patients who had other indications for prolonged intravenous antibiotics, suspected reduced gastrointestinal uptake, or a body mass index (BMI) > 40 were excluded.

Those in the oral limb had antibiotics which had moderate to high bioavailability and were given two antibiotics with different mechanisms of action and metabolism to reduce the risk of de facto monotherapy. The POET trial showed, in select stable patients with left-sided infective endocarditis, that changing to oral antimicrobial regimens was noninferior to continued intravenous antimicrobial regimens. A total of 139 patients (35%) had at least one major coexisting medical condition which was equally distributed between the two groups, 67 (17%) had diabetes, 46 (12%) had renal failure, 28 (7%) were on dialysis and 13 (3%) had liver disease. The most frequently identified pathogen was *Streptococcus*, spp., followed by *Staphylococcus aureus* and *Enterococcus faecalis*. The aortic valve was affected in the majority of cases, and in 107 patients (27%), a previously inserted prosthetic valve was affected. The composite primary outcome of all-cause mortality, unplanned cardiac surgery, embolic event or relapse of bacteraemia with the primary pathogen occurred in 9.0% (22/201) in the oral limb and 12.1% (24/199) in the intravenous limb, meeting the criterion for non-inferiority. There were fewer all-cause mortalities in the oral limb 3.5% (7/201) than the intravenous limb 6.5% (13/199), the cause of which was not clear. Adverse effects from antibiotics were reported in 22 patients (6%), with 12 patients (6%) in the intravenously treated limb and 10 (5%) in the orally treated limb. The most frequent adverse effects were allergy (50%), bone marrow suppression (27%) and gastrointestinal effects (14%), which highlights the importance of close monitoring of patients treated with OPAT. Hybrid intravenous/oral antimicrobial regimens to treat infective endocarditis within the outpatient setting can have particular benefits in selected patients with a history of intravenous drug use and those who have difficult venous access and can further reduce healthcare resources with patients self-administering their own antibiotics.

### 3.7. Monitoring during OPAT

During the OPAT program patients should be monitored regularly. Weekly laboratory investigations should be performed, monitoring patients’ renal and hepatic function and ensuring a decrease in/normalization of inflammatory markers (leucocytes, c-reactive protein) [4]. Close monitoring of circulating levels of aminoglycosides and glycopeptides is vital to ensure patients on these antimicrobials have appropriate dose adjustments to avoid inefficacy or toxicity [19,25]. The AHA [3] recommends repeating TTE and/or TEE for re-evaluation of patients with IE who have a change in clinical signs or symptoms.

### 3.8. Follow-Up after Completion of OPAT

Patients require ongoing monitoring after completion of their antimicrobials as most post-endocarditis treatment complications occur within the first 12 months. Recurrence of infective endocarditis is estimated to be 2–6% within the first year. Therefore, follow-up with TTE and blood testing for inflammatory markers at 1, 3, 6 and 12 months is recommended [1,7]. Preventative measures and patient education on good dental hygiene, avoidance of intravenous drug use and high-risk body piercings/tattoos and consideration of antibiotic prophylaxis with dental and other invasive procedures is essential to minimize risk [1,3,7,16].

## 4. Conclusions

**Figure 1:**
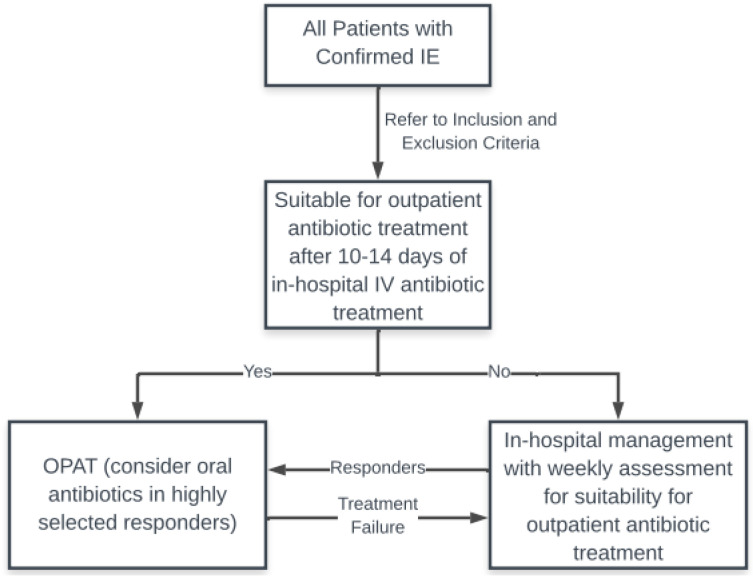
Suggested OPAT pathway. IE: infective endocarditis; IV: intravenous.

Infective endocarditis is an infectious disease with significant morbidity and mortality which has not improved over the last few decades. This infection requires a prolonged course of intravenous antibiotic therapy often for a period of 4–6 weeks and therefore contributes a large economic and resource burden to the healthcare system. OPAT in selected patients is safe and beneficial for both the patient and the healthcare system. A developed model of care with a multidisciplinary OPAT team is essential for the success and safe administration of an outpatient program. We propose that healthcare systems develop pathways for OPAT in IE patients, taking into consideration the available resources, and that all patients with IE be screened regularly for their suitability for outpatient management (Figure 1 [18]).

## Figures and Tables

**Table 1:** Summary of Studies of Outpatient Parenteral Antibiotic Therapy (OPAT) in Infective Endocarditis [18].

Study	Study Location	Study Type	Number of IE Episodes	Mean age Male:Female *	Readmissions during Treatment	Mortality	Main Findings
Pajarόn et al. 2015 [4]	Spain	Retrospective and prospective	48	63.1 34:11	6 (12.5%)	5 (10.4%) at 1 year	Self-administered OPAT is at least as effective in terms of efficacy and safety as healthcare-professional-administered OPAT.
Larioza et al. 2009 [5]	United States of America	Retrospective	43	N/A 29:14	10 (23.3%)	0 (0%) at 1 year	Patients completed at least 66% of their total treatment duration as outpatients after an inpatient stabilisation period (typically 1–2 weeks).
Lacroix et al. 2014 [11]	France	Retrospective	18	59.5 11:7	3 (16.7%)	1 (5.6%) at 3 months	OPAT in selected patients seems effective, safe and reduces costs by approximately EUR 15,000 per patient.
Cervera et al. 2011 [12]	Spain	Prospective	73	59.5 55:18	12 (16.4%)	3 (4.1%) at 1 year	OPAT for IE could be a safe and efficacious therapeutic option for carefully selected patients.
Partridge et al. 2012 [13]	United Kingdom	Retrospective	36	54.7 27:7	5 (13.9%)	1 (2.8%) at 30 months	OPAT is safe and effective in the management of IE, including for some patients who would have previously been considered high risk of complications (IDSA guidelines), such as those with infected prosthetic valves and *Staphylococcus aureus* IE.
Htin et al. 2013 [19]	Australia	Retrospective	68	Median: 68 59:9	3 (4.4%)	2 (2.9%) at 1 year	OPAT in IE is safe and effective, including prosthetic valve infections and those who have undergone valve replacement surgery. Caution in patients with *Staphylococcus aureus* IE.
Chirillo et al. 2013 [20]	Italy	Prospective	292	57.4 190:102	N/A	35 (34%) before and 31 (16%) after intervention of an OPAT team	Comparing the outcomes of patients with IE prior to (1996–2002) and after (2003–2009) the introduction of a formalised multidisciplinary OPAT team. Reveals a significant reduction in overall mortality.
McMahon et al. 2008 [21]	Australia	Multi-centre prospective	40	56.5 30:10	3 (7.5%)	N/A	Hospital-in-the-home treatment is safe and effective. Caution in patient selection is required for *Staphylococcus aureus* IE.
Iversen et al. 2018 (POET trial) [22]	Denmark	Multi-centre, randomised, unblinded, non-inferiority	400 (199 intravenous, 201 oral treatment)	67 308:92	N/A	20 (5%) at 6 months	In selected patients, a shift from intravenously administered to orally administered antibiotic treatment was non-inferior to continued intravenous antibiotic treatment.

* Some patients had multiple episodes of infective endocarditis.

**Table 2:** Patient Selection for OPAT.

**General OPAT Criteria**
- Adequate cognitive function and stable mental health
- Access to outpatient healthcare services (clinics/HITH)
- Access to transport if required
- Telephone access
- Ability of the healthcare system to provide daily review if required
**Patient Criteria**
- Absence of active illicit drug use
- Caution with high-risk patients (e.g., elderly, prosthetic endocarditis, multiple patient comorbidiities)
- Caution with high-risk culprit organisms (e.g., ***Staphylococcal aureus*, *fungi*** and ***non-HACEK Gram-negative bacilli***)
- Absence of infective endocarditis complications (e.g., heart failure, renal failure, septic shock, neurological complications)
- Absence of treatment complications (e.g., adverse drug effects, diarrhoea, nausea, vomiting and catheter line infections) - Stable intravenous access
- Absence of uncontrolled extra-cardiac foci of infection
**Laboratory Criteria**
- Decreasing inflammatory markers (neutrophil count, CRP) - Stable renal function (GFR, creatinine) and hepatic function (LFTs, albumin, INR)
**Electrocardiogram and Echocardiogram criteria**
- Absence of conduction block (2nd and 3rd degree AV block)
- Decrease in size of the vegetation since starting in-hospital therapy
- Absence of para-valvular complications
- Vegetation ≤10 mm
**Without Indications for Surgery**
- Aortic or mitral IE with severe acute regurgitation causing refractory pulmonary oedema/shock - Aortic or mitral IE with fistula into a cardiac chamber/pericardium causing refractory pulmonary oedema/shock - Locally uncontrolled infection (e.g., abscess, false aneurysm, enlarging vegetation, persisting fever and positive blood culture for ≥10 days) - Infection caused by fungi or multi-resistant microorganisms - Prevention of embolism with a large vegetation >10 mm resulting in complications (embolic episode, heart failure, persistent infection, abscess) - Prevention of embolism with a large vegetation >15 mm

HITH: hospital in the home; CRP: c-reactive protein; GFR: glomerular filtration rate; LFTs: liver function tests; INR: international normalised ratio; AV block: atrioventricular block.
